# Comprehensive assessment of dietary micronutrient profiles and their effects on hemoglobin levels and anemia: provincial nutrition and health monitoring

**DOI:** 10.3389/fnut.2025.1638705

**Published:** 2025-11-26

**Authors:** Yan Wang, Yun Lin, Ye Lv, Yiyuan Jin, Hua Gao, Sihai Gao, Pingping Shentu, Chao Xing, Yan Chen, Shiguang Zhao, Ying Cong, Guangtao Liu, Peiwei Xu, Ronghua Zhang

**Affiliations:** 1 Zhejiang Provincial Center for Disease Control and Prevention, Hangzhou, Zhejiang, China; 2 Jiaxing Center for Disease Control and Prevention, Jiaxing, Zhejiang, China; 3 Hangzhou Center for Disease Control and Prevention (Hangzhou Institute for Health Supervision), Hangzhou, Zhejiang, China; 4 Taizhou Center for Disease Control and Prevention (Taizhou Health Inspection Institute), Taizhou, Zhejiang, China; 5 Ningbo Center for Disease Control and Prevention, Ningbo, Zhejiang, China; 6 Wenzhou Center for Disease Control and Prevention (Wenzhou Institute of Public Health Supervision), Wenzhou, Zhejiang, China; 7 Jinhua Center for Disease Control and Prevention, Jinhua, Zhejiang, China; 8 Shaoxing Center for Disease Control and Prevention, Shaoxing, Zhejiang, China; 9 Zhoushan Center for Disease Control and Prevention, Zhoushan, Zhejiang, China; 10 Quzhou Center for Disease Control and Prevention, Quzhou, Zhejiang, China; 11 Lishui Center for Disease Control and Prevention, Lishui, Zhejiang, China; 12 Huzhou Center for Disease Control and Prevention, Huzhou, Zhejiang, China

**Keywords:** micronutrients, vitamin C, selenium, hemoglobin, anemia

## Abstract

**Background:**

Micronutrients play a major role in regulating public health. Inadequate intake of micronutrients is a frequent and potentially hazardous occurrence that may lead to the development of common pathologies, such as anemia, a widespread health concern. This study aimed to estimate dietary micronutrient intake inadequacies and to investigate the association between micronutrients and hemoglobin (Hb) levels or anemia.

**Methods:**

This study employed a cross-sectional design, including 15,810 participants. The questionnaire used in this study consisted of sections on demographic characteristics, lifestyles, dietary assessment, and disease history. Dietary intake information was collected using the 3-day 24-h dietary recall method. Hemoglobin levels were measured using the colorimetric method. The individual effect of micronutrients on Hb and anemia was evaluated using restricted cubic splines (RCS) and multiple logistic regression. The combined effect was assessed by weighted quantile sum regression (WQSR).

**Results:**

The study included 7,570 male and 8,240 female participants from a representative survey of Zhejiang Province, China. The prevalence of anemia was 12.2%. The mean Hb level in the overall population was 139 (15.7) g/L. Specifically, the mean Hb level was 142 (12.8) g/L in normal participants and 114 (11.2) g/L in anemic participants. Inadequate dietary intake rate for riboflavin (88.1%), vitamin E (87.6%), and calcium (81.6%) was high, while the rate of inadequate dietary intake for iron (13.3%), iodine (7.06%), and selenium (3.15%) was low. The influencing factors of anemia included age, sex, living area, income, smoking status, physical activity, body mass index (BMI), and diabetes. A linear dose–response positive relationship between thiamin, vitamin C, and selenium and Hb was found among women. According to the results of multiple logistic regression, thiamin [odds ratio (OR): 0.81; 95% confidence interval (CI): 0.67–0.97] and selenium (OR: 0.93; 95% CI: 0.88–0.99) were significantly associated with a decreased risk of anemia among women.

**Conclusion:**

The prevalence of inadequate micronutrient intake was generally high, and micronutrient intakes had a significant protective effect against anemia, suggesting that interventions should be conducted to overcome the micronutrient intake inadequacies.

## 1 Introduction

Micronutrients, which include both vitamins and minerals, are vital components of a balanced diet (1), as they play indispensable roles in a plethora of physiological processes within the human body (2). Inadequate intake of essential micronutrients, such as iron, zinc, and vitamin A, among others, can lead to micronutrient deficiencies. A properly functioning immune system needs an adequate supply of micronutrients to safeguard the cells involved in the innate immune response from damage and to repair tissues damaged by the host defense against infectious agents (3–5). Micronutrient deficiencies can, therefore, increase disease morbidity and mortality, thus significantly impairing human potential on a global scale (6). Estimates of inadequate micronutrient intakes are needed to fully comprehend the burden of micronutrient malnutrition.

Micronutrient deficiencies are one of the most common etiologies of anemia (7, 8), a widespread global health concern associated with negative health impacts and substantial health and economic costs (9). Symptoms of anemia in adults include weakness, difficulty concentrating, and reduced physical work capacity (10). In older adults, anemia can decrease quality of life, impair cognitive function, and increase mortality (8). Anemia is a pathophysiologically diverse and often multifactorial condition (11). Except for iron (12), research on the intake of other micronutrients (including vitamins and minerals) and anemia among the general population remains limited. Further investigations are needed to explore the relationship between multiple micronutrients and anemia, especially regarding exposure-response relationships and their overall effects.

The present study aimed to explore representative data retrieved from the 2023 Provincial Nutrition and Health Survey of Zhejiang Province, China, to obtain comprehensive estimate of the level of micronutrient intake across the province, including six vitamins (vitamin A, thiamin, riboflavin, niacin, vitamin C, and vitamin E) and six minerals (calcium, iron, zinc, selenium, magnesium, and iodine). As a further goal, we also endeavored to evaluate the individual and combined effect of micronutrients on hemoglobin (Hb) levels and anemia in this population.

## 2 Methods

### 2.1 Schematic overview of the survey program

**Figure 1:**
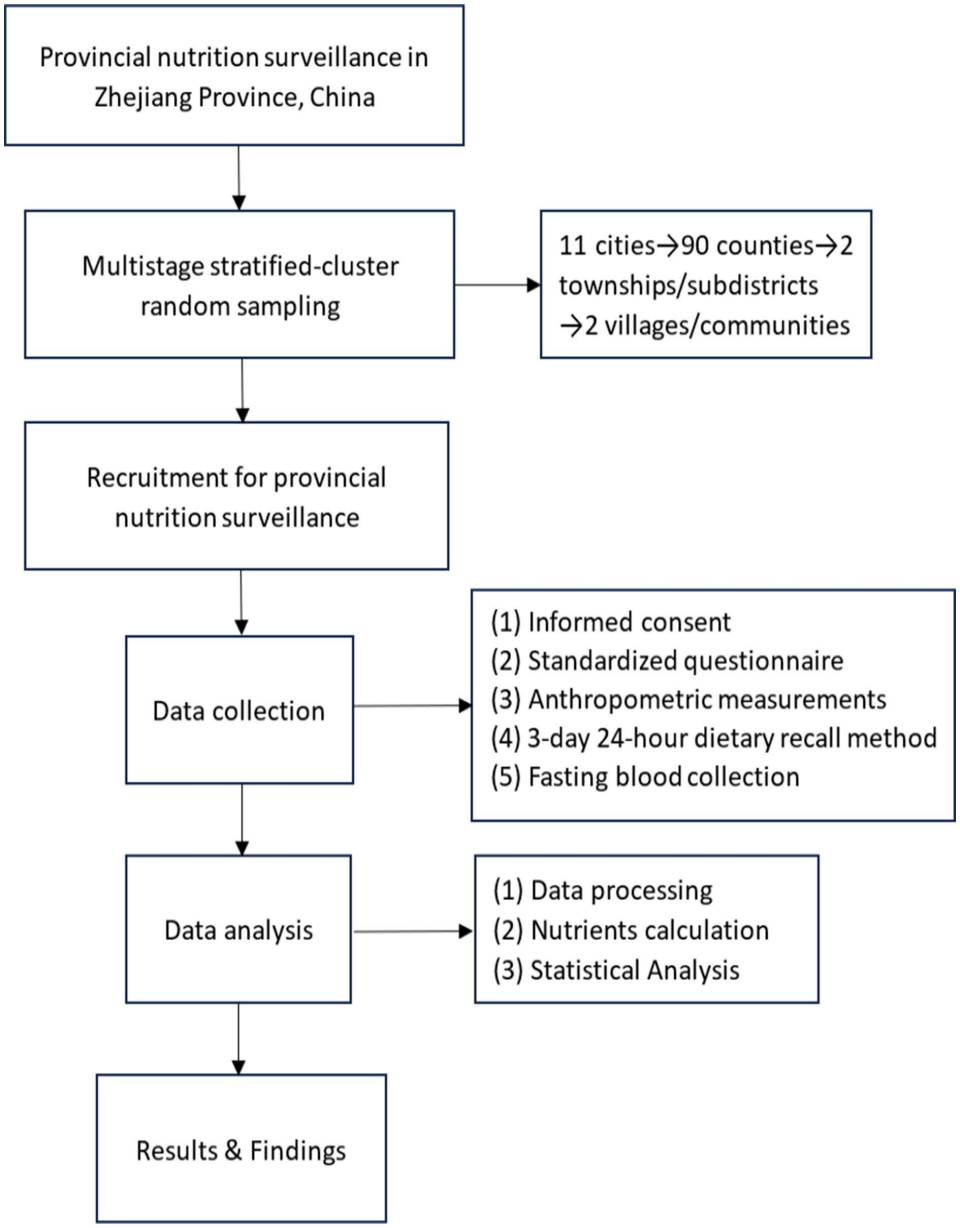
Flow diagram of the study procedures.

A schematic flow diagram illustrating the core procedures in this study is presented in Figure 1. This flow diagram systematically maps the operational framework designed to achieve the core objectives of this study, which are to investigate the prevalence of anemia among adults in Zhejiang Province and to explore the association between micronutrient intake and anemia. The rationale for this investigation is grounded in the need to inform targeted public health strategies for anemia prevention, given its status as a significant nutritional concern and its potential links with micronutrient intake. The research instruments, including the standardized questionnaire and the detailed dietary assessment protocol, were adopted from established surveys, such as the China Health and Nutrition Survey (CHNS), a national nutrition survey (13, 14), to ensure the comprehensive and accurate collection of demographic, lifestyle, and dietary data. All procedures implemented in this study, from participant recruitment and data collection to sample handling, were rigorously designed and conducted to safeguard the rights, safety, and well-being of every participant. The study protocol received full approval from the Institutional Ethical Committee of the Institute of Nutrition and Health, Zhejiang Provincial Center for Disease Control and Prevention (Approval number: 2023–002-01).

### 2.2 Study population

The present study included participants aged 18–102 years who were recruited for provincial nutrition surveillance in Zhejiang Province, China, in 2023. The provincial nutrition surveillance employed multistage stratified-cluster random sampling to select a representative study population from 90 counties (cities and districts) across 11 cities (Supplementary Figure S1). Subsequently, two townships or subdistricts were randomly chosen from each monitoring county, followed by the random selection of two villages or communities from the townships or subdistricts. The numbers of people in each age group were evenly distributed between urban and rural areas as well as between male and female participants. More details can be found in previous studies (15, 16). Participants were included if they (1) were aged ≥ 18 years; (2) were not pregnant; and (3) had available data on dietary intake and Hb testing. A total of 15,810 subjects were included in the analysis.

The study was conducted in accordance with the tenets of the Declaration of Helsinki and was approved by the Ethics Committee of the Institute of Nutrition and Health, Zhejiang Provincial Center for Disease Control and Prevention (Approval number: 2023–002-01). Informed consent was obtained from all participants before the onset of the study.

### 2.3 Data collection and dietary assessment

Information about the participants, including demographic characteristics, lifestyle, and disease history, was obtained through face-to-face interviews using a standardized questionnaire. The demographic variables included sex (male or female), age, education (primary school and below, middle school, or senior high school and above), and income. The annual per capita household income was classified into three levels based on the 33rd percentile and 67th percentile: low-income, middle-income, and high-income. Lifestyle data consisted of smoking status (classified as never, ever, and current), physical activity, and body mass index (BMI). In this study, data on physical activity were collected using the short form of the International Physical Activity Questionnaire (IPAQ). The level of physical activity of a specific intensity performed by an individual per week was calculated as follows: the Metabolic Equivalent of Task (MET) value corresponding to that intensity of physical activity × weekly frequency (days/week) × daily duration (min/day). The total physical activity level was calculated by summing the levels of the three intensities of physical activity: walking, moderate-intensity physical activity, and vigorous-intensity physical activity. Physical activity was categorized into low, moderate, and high levels in accordance with the standards of the IPAQ (17).

Weight and height were measured using a calibrated electronic scale (accuracy: 0.1 kg) and a stadiometer (accuracy: 0.1 cm), with participants in a fasting state, wearing light clothing, and without shoes. Body mass index was calculated by dividing body weight (kg) by the square of the height (m^2^). The values obtained were then categorized as follows: low (<18.5 kg/m^2^), normal (18.5–24.0 kg/m^2^), overweight (24.0–28.0 kg/m^2^), and obese (≥28 kg/m^2^) according to the Working Group on Obesity in China.

In this study, blood pressure was measured using an automatic electronic sphygmomanometer (HEM-7130, Omron, Japan), following the American Heart Association’s standardized protocol (18). Additionally, blood pressure was measured three times at 30-s intervals in the sitting position after the subjects had rested for 5 min. The mean values of the measurements were used for analysis. Hypertension was defined as having a prior diagnosis of hypertension or having a measured BP ≥ 140/90 mm Hg or the use of antihypertensive treatment during the last 2 weeks.

After overnight fasting, venous blood samples were collected from the participants and processed by centrifugation for separation. Meanwhile, fasting blood glucose levels were measured using the hexokinase method with an automatic biochemical analyzer (LABOSPECT 008 AS, Hitachi, Tokyo, Japan). Diabetes was defined as fasting blood glucose ≥7.0 mmol/L or having a self-reported previous diagnosis of diabetes from a physician or self-reported treatment with insulin or antidiabetic medications during the last 2 weeks. High-sensitivity C-reactive Protein (Hs-CRP) was measured using the immunoturbidimetric method with the same automatic biochemical analyzer (LABOSPECT 008 AS, Hitachi, Tokyo, Japan).

This study employed the 3-day consecutive 24-h dietary recall method for dietary assessment. A standardized dietary questionnaire identical to that utilized in the CHNS was adopted (13, 14). During the visits, dietary information on breakfast, lunch, dinner, and extra meals, snacks, and condiments was collected. Dietary intake was measured by three consecutive 24-h dietary recalls from participants in combination with a weighing inventory taken over the same 3 days at the household level to record the amount of food consumed (including meals outside). The survey period included 2 weekdays and 1 weekend day to better reflect usual dietary patterns. This questionnaire covers comprehensive information, including not only the core 3-day consecutive dietary recall data—recording the types and consumption of all foods ingested by participants daily, such as staple foods, meat, eggs, dairy products, fruits, vegetables, and snacks—but also two key supplementary surveys: first, the 3-day consecutive weighing record of household cooking oil and condiments (e.g., salt, soy sauce, and sugar), requiring participants to accurately weigh and record the total daily usage of cooking oil, salt, and other condiments during household cooking; second, the recording of household dining population, which involves documenting the actual number of people—including adults and children, with children converted to adult dining equivalents based on age—who ate together in the household each day during the survey period, so as to adjust the individual actual intake of food shared within the household.

The standard tableware and food atlas were used to assist the investigation. Portions of individually consumed foods (e.g., snacks and purchased items) were estimated via dietary recall, using common containers (bowls and plates) or physical references. For family-shared foods, individual intake was calculated by combining total cooked amounts, household dining population (children converted to adult equivalents), and self-reported consumption proportions. Household total consumption of cooking oil and condiments over 3 days was derived from weighing records (initial amount + additions − remaining amount) and then allocated to individuals by dining population. Nutrient intake per person was calculated based on the China Food Composition Tables published in 2018 (19). The daily intake of a certain nutrient was calculated by multiplying the edible portion of each food by the nutrient content and then summing up the nutrient intake of all foods. The nutrient contents of each food were obtained from the China Food Composition Tables by food codes. Implausible data points, such as an energy intake <500 kcal or >5,000 kcal per day, were excluded. The probability method was used to calculate the dietary inadequate intake rate of micronutrients (20, 21), according to the estimated average requirement (EAR).

### 2.4 Definition of anemia

Fasting venous blood was collected from the participants on the morning of the physical examination day, and hematological and biochemical parameters (blood glucose and Hs-CRP) were analyzed on the same day. Hb levels were measured using the colorimetric method by a hematology analyzer (BC-6800 PLUS, Mindray, Shenzhen, China). The World Health Organization released the guideline on Hb cut-offs to define anemia in individuals and populations, aiming to apply a consistent, evidence-informed approach to support health providers and policy makers to implement anemia detection (22, 23). Anemia was defined as an Hb concentration <120 g/L for women (not pregnant) or <130 g/L for men, according to World Health Organization guidelines (22, 23). Applying the criteria was crucial for two primary reasons. First, it ensures the comparability of our findings on anemia prevalence and associated risk factors with other regional, national, and international studies, which universally adopt this standard (24, 25). Second, using this established, evidence-based benchmark provides objective and actionable data for public health planning, enabling the development of targeted interventions for anemia prevention in Zhejiang Province.

### 2.5 Statistical analysis

Descriptive statistics for the demographic characteristics and lifestyles of the participants were calculated. Continuous variables were presented as means and standard deviations (SDs) and compared using the Mann–Whitney U test, while categorical variables were presented as frequencies and proportions and compared using the chi-squared test. The normality of each micronutrient, Hb, intake of energy, and Hs-CRP was examined using the Shapiro–Wilk test, and the levels of micronutrients, Hb, intake of energy, and Hs-CRP were natural log (ln)-transformed for further analyses.

A multiple logistic regression analysis was used to assess the association between factors and anemia, exploring the potential correlates of anemia. Factors included age, sex (male or female), living area (urban or rural), BMI (low, <18.5 kg/m^2^; normal, 18.5–24.0 kg/m^2^; overweight, 24.0–28.0 kg/m^2^; or obese, ≥28 kg/m^2^), education level (primary school and below, middle school, or senior high school and above), income (low, middle, and high), smoking status (never, ever, or current), physical activity (low, moderate, or high), use of dietary supplement (yes or no), Hs-CRP levels, and medical conditions [hypertension (no or yes) and diabetes (no or yes)]. A multiple logistic regression analysis was also used to evaluate the associations between micronutrient intake levels and anemia, and the results were expressed as odds ratios (ORs) and 95% confidence intervals (95% CIs). Restricted cubic splines (RCS) in linear regression were conducted to explore the dose–response relationships between the micronutrient levels and Hb with three knots (5th, 50th, and 95th percentiles).

The weighted quantile sum regression (WQSR) analyses were conducted to evaluate the association between micronutrient mixture and Hb or anemia. The WQSR analyses were conducted to reduce the high dimensionality and multicollinearity by combining multiple correlated components into weighted indices. The weights of micronutrients range from 0 to 1, and the sum is 1 (26). WQSR was performed by the “gWQS” package (R version 4.4.2). The number of bootstrap samples used to estimate the parameters was set to 1,000. A total of 60% of the dataset was used for validation. The WQSR model provides a unidirectional evaluation of mixture effects. Both positive and negative directions were analyzed in the present study.

A *p*-value of <0.05 was considered statistically significant. All analyses were performed with R software (version 4.4.2) and SAS version 9.4 (SAS Institute, Cary, NC, United States).

## 3 Results

### 3.1 Basic characteristics of participants

The population demographics of the participants are shown in Table 1. A total of 15,810 participants (7,570 men and 8,240 women) were included in the analyses. Of those, 1,933 (12.2%) were identified as having anemia, with a 10% prevalence for men and 14.2% for women. The mean age in the overall population was 54.9 (15.4) years. Specifically, the mean age was 53.5 (15.2) years in normal participants and 56.4 (16.6) years in anemic participants. The mean Hb level in the overall population was 139 (15.7) g/L. Specifically, the mean Hb level was 142 (12.8) g/L in normal participants and 114 (11.2) g/L in anemic participants. Compared with participants without anemia, those with anemia were older, had lower Hb levels, were more often female, more likely to live in rural areas, had lower education and income levels, were less likely to be current smokers, and were less frequently overweight and obese. The participants with anemia were also more likely to have hypertension and diabetes. Participants with anemia also showed higher levels of Hs-CRP.

**Table 1:** Characteristics of participants (*n* = 15,810).

Variables	Normal (*n* = 13,877)	Anemia (*n* = 1,933)	*p*-value
Age (years), mean (SD)	53.5 (15.2)	56.4 (16.6)	<0.001
Hb levels (g/L), mean (SD)	142 (12.8)	114 (11.2)	<0.001
Sex			<0.001
Male	6,811 (49.1%)	759 (39.3%)	
Female	7,066 (50.9%)	1,174 (60.7%)	
Living area			0.005
Urban	6,673 (48.1%)	863 (44.6%)	
Rural	7,204 (51.9%)	1,070 (55.4%)	
Education level			<0.001
Primary school and below	4,882 (35.2%)	816 (42.2%)	
Middle school	3,637 (26.2%)	480 (24.8%)	
Senior high school and above	5,358 (38.6%)	637 (33.0%)	
Annual per capita income			<0.001
Low	4,152 (29.9%)	675 (34.9%)	
Middle	4,214 (30.4%)	582 (30.1%)	
High	5,511 (39.7%)	676 (35.0%)	
Smoking status			<0.001
Never	10,510 (75.7%)	1,582 (81.8%)	
Ever	1,029 (7.40%)	178 (9.20%)	
Current	2,338 (16.8%)	173 (8.90%)	
Physical activity			0.327
Low	10,737 (77.4%)	1,523 (78.8%)	
Moderate	2,910 (21.0%)	383 (19.8%)	
High	230 (1.60%)	27 (1.40%)	
BMI			<0.001
Low	534 (3.80%)	129 (6.70%)	
Normal	7,120 (51.3%)	1,187 (61.4%)	
Overweight	4,795 (34.6%)	510 (26.4%)	
Obese	1,428 (10.3%)	107 (5.50%)	
Hypertension			0.034
No	10,215 (73.6%)	1,379 (71.3%)	
Yes	3,662 (26.4%)	554 (28.7%)	
Diabetes			0.002
No	12,684 (91.4%)	1,726 (89.3%)	
Yes	1,193 (8.60%)	207 (10.7%)	
Use of dietary supplements			
No	13,320 (96.0%)	1,844 (95.4%)	0.219
Yes	557 (4.00%)	89 (4.60%)	
Hs-CRP (mg/L), mean (SD)	1.52 (3.10)	1.89 (4.71)	<0.001

SD, standard deviation; BMI, body mass index.

### 3.2 Levels of micronutrients and the rate of intake inadequacies

**Figure 2:**
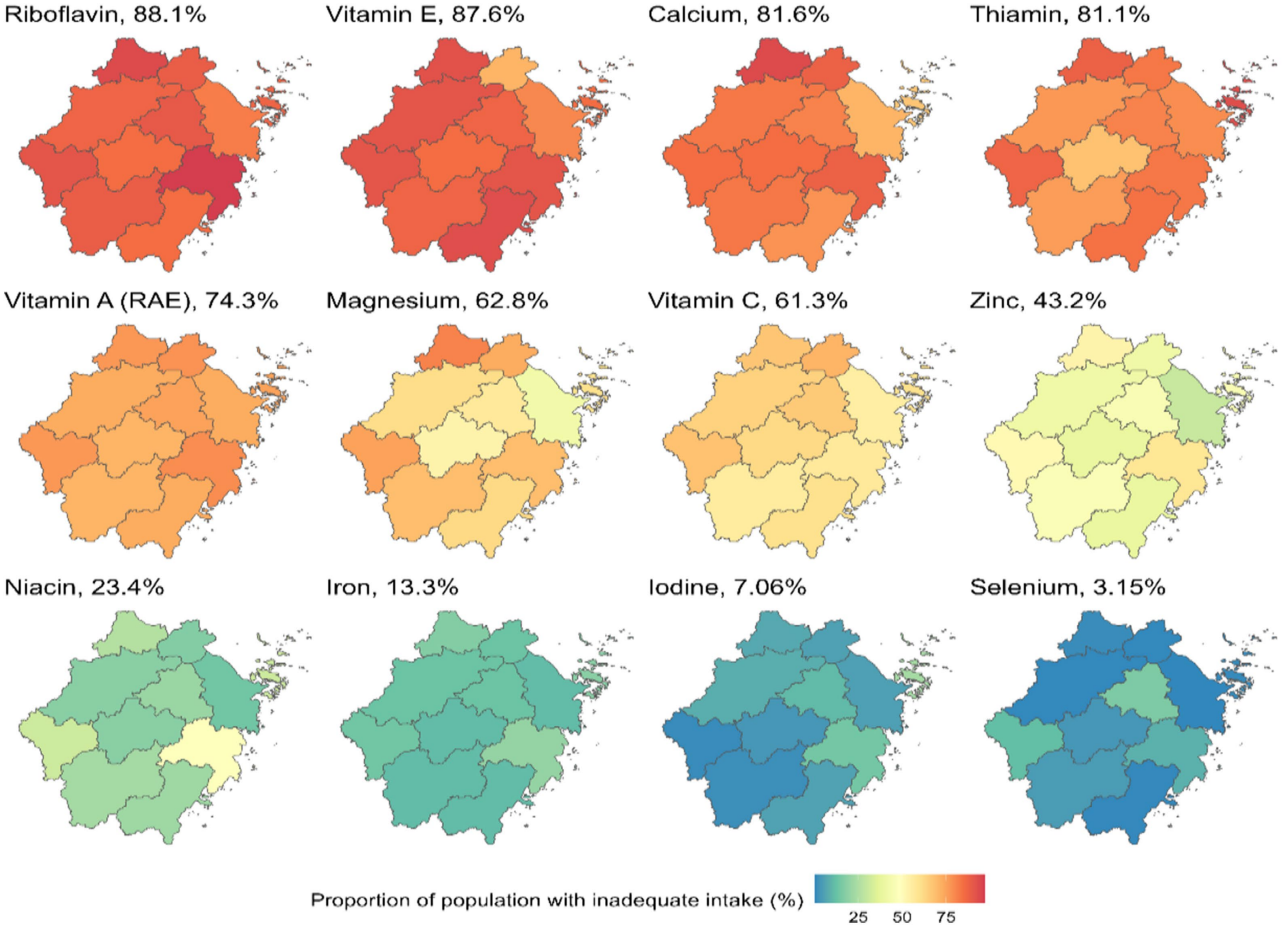
Estimated rate of intake inadequacies by city and nutrient. RAE, retinol activity equivalents.

The levels of micronutrients are presented in Table 2. Compared with the participants without anemia, those with anemia had lower levels of thiamin, niacin, zinc, selenium, and magnesium. In addition, the inadequacy rates of micronutrient intake were generally high in our population (Figure 2). Inadequate dietary intake rate for riboflavin (88.1%), vitamin E (87.6%), and calcium (81.6%) was high, while the rate of inadequate dietary intake for iron (13.3%), iodine (7.06%), and selenium (3.15%) was low.

**Table 2:** Levels of micronutrient intake (median and IQR) (*n* = 15,810).

Micronutrients	Overall	Normal	Anemia	*p*-value
Vitamin A (μg RAE/day)	294 (197, 423)	294 (198, 423)	288 (189, 418)	0.326
Thiamin (mg/day)	0.78 (0.58, 1.05)	0.79 (0.59, 1.05)	0.75 (0.55, 1.02)	**0.001**
Riboflavin (mg/day)	0.67 (0.51, 0.88)	0.67 (0.51, 0.89)	0.66 (0.50, 0.88)	0.259
Niacin (mg/day)	12.9 (9.97, 17.0)	13.0 (10.0, 17.0)	12.6 (9.66, 16.8)	**0.004**
Vitamin C (mg/day)	64.4 (39.7, 99.1)	64.6 (39.9, 99.7)	62.9 (38.6, 95.0)	0.058
Vitamin E (mg/day)	7.02 (5.00, 9.78)	7.04 (5.01, 9.81)	6.89 (4.88, 9.57)	0.084
Calcium (mg/day)	390 (271, 554)	391 (270, 556)	385 (275, 544)	0.697
Iron (mg/day)	15.1 (11.4, 20.5)	15.2 (11.4, 20.5)	15.1 (11.3, 20.5)	0.476
Zinc (mg/day)	8.57 (6.58, 11.3)	8.60 (6.61, 11.3)	8.39 (6.40, 11.1)	**0.028**
Selenium (μg/day)	52.3 (33.4, 99.7)	52.6 (33.6, 101)	49.2 (31.9, 91.5)	**0.004**
Magnesium (mg/day)	230 (179, 295)	230 (179, 296)	225 (175, 288)	**0.009**
Iodine (μg/day)	136 (54.6, 226)	136 (54.6, 224)	139 (53.8, 240)	0.231

Bold values indicate a statistically significant difference at *p* < 0.05.

### 3.3 Determinants of anemia

Increasing age, being female, living in a rural area, and diabetes were significantly and positively associated with anemia. High income, current smoking status, moderate physical activity, and being overweight or obese were negatively associated with anemia (Table 3).

**Table 3:** Logistic regression analysis of the correlates of anemia (*n* = 15,810).

Variables	Anemia	*p*-value
Age (years)	1.01 (1.01, 1.02)	<0.001
Sex		
Male	Reference	
Female	1.33 (1.18, 1.50)	<0.001
Living area		
Urban	Reference	
Rural	1.15 (1.04, 1.27)	0.006
Education level		
Primary school and below	Reference	
Middle school	0.99 (0.87, 1.13)	0.857
Senior high school and above	1.03 (0.88, 1.20)	0.755
Annual per capita income		
Low	Reference	
Middle	0.91 (0.81, 1.03)	0.144
High	0.83 (0.73, 0.93)	0.002
Smoking status		
Never	Reference	
Ever	1.22 (1.01, 1.47)	0.037
Current	0.59 (0.49, 0.71)	<0.001
Physical activity		
Low	Reference	
Moderate	0.83 (0.73, 0.94)	0.003
High	0.78 (0.52, 1.17)	0.231
BMI		
Low	1.46 (1.19, 1.80)	<0.001
Normal	Reference	
Overweight	0.63 (0.56, 0.71)	<0.001
Obese	0.45 (0.37, 0.56)	<0.001
Hypertension		
No	Reference	
Yes	1.04 (0.92, 1.17)	0.579
Diabetes		
No	Reference	
Yes	1.19 (1.01, 1.41)	0.038
Use of dietary supplements		
No	Reference	
Yes	1.17 (0.93, 1.48)	0.185
Hs-CRP	0.98 (0.94, 1.03)	0.357

### 3.4 Relationships of individual micronutrients with Hb and anemia

The associations between micronutrient intake and anemia among all participants and among male and female participants are shown in Table 4. Covariates included age, sex, energy intake, living area, education level, income, smoking status, physical activity, BMI, hypertension, diabetes, use of dietary supplements, and Hs-CRP level. Every 1-unit increase in the ln-transformed levels of selenium was significantly associated with a 7.00% decrease in anemia risk among women (OR: 0.93; 95% CI: 0.88, 0.99; *p* = 0.021). Every 1-unit increase in the ln-transformed levels of thiamin was significantly associated with a 19.0% decrease in anemia risk among women (OR: 0.81; 95% CI: 0.67, 0.97; *p* = 0.024).

**Table 4:** Association between intake of micronutrients and anemia (OR and 95% CI).

Micronutrients	Overall	Male	Female
Vitamin A	1.02 (0.95, 1.10)	1.07 (0.96, 1.20)	0.99 (0.90, 1.09)
Thiamin	0.88 (0.76, 1.02)	1.03 (0.82, 1.29)	**0.81 (0.67, 0.97)**
Riboflavin	1.14 (0.99, 1.31)	1.23 (0.99, 1.52)	1.09 (0.92, 1.30)
Niacin	1.04 (0.89, 1.22)	1.10 (0.85, 1.41)	1.02 (0.83, 1.24)
Vitamin C	0.97 (0.90, 1.03)	0.97 (0.87, 1.08)	0.97 (0.89, 1.05)
Vitamin E	0.99 (0.90, 1.10)	0.95 (0.82, 1.12)	1.03 (0.90, 1.17)
Calcium	1.09 (0.99, 1.19)	1.15 (0.99, 1.33)	1.05 (0.93, 1.18)
Iron	1.10 (0.96, 1.25)	1.11 (0.90, 1.36)	1.09 (0.92, 1.29)
Zinc	1.08 (0.92, 1.27)	1.19 (0.92, 1.55)	1.01 (0.82, 1.25)
Selenium	0.98 (0.94, 1.02)	1.04 (0.97, 1.12)	**0.93 (0.88, 0.99)**
Magnesium	0.93 (0.78, 1.10)	0.91 (0.69, 1.20)	0.94 (0.75, 1.18)
Iodine	1.01 (0.98, 1.05)	1.01 (0.95, 1.06)	1.02 (0.98, 1.07)

Bold values indicate a statistically significant difference at *p* < 0.05.

Adjusted by age, sex (for the overall population), energy intake, living area, education level, income, smoking status, physical activity, BMI, hypertension, diabetes, use of dietary supplements, and Hs-CRP level.

**Figure 3:**
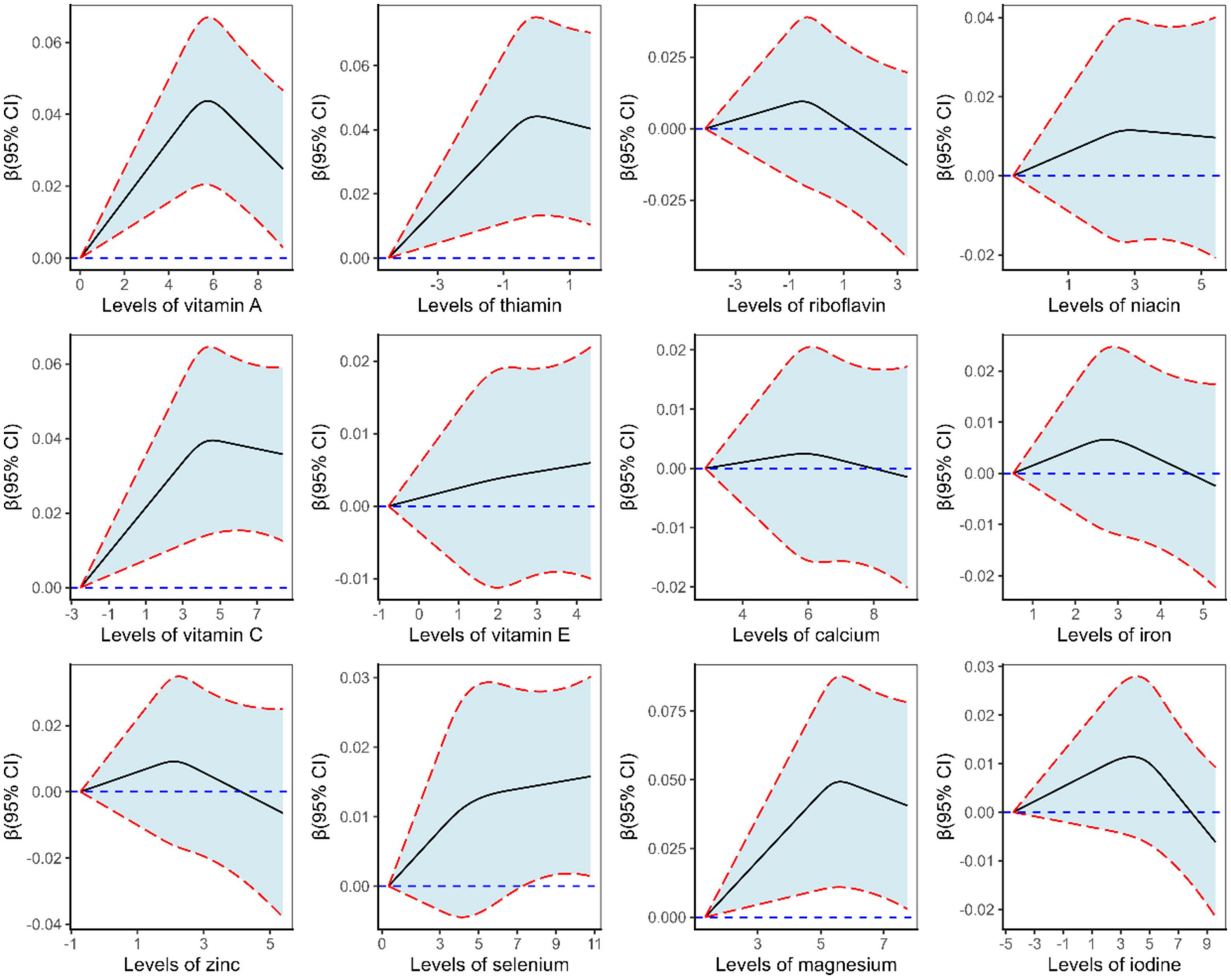
*β* (solid lines) and 95% confidence intervals (CIs, dashed lines) of the ln-transformed level of micronutrient for Hb level from restricted cubic splines in the total population.

By adjusting for covariates (age, sex, energy intake, living area, education level, income, smoking status, physical activity, BMI, hypertension, diabetes, use of dietary supplements, and Hs-CRP level), the linear associations between micronutrient intakes and Hb were explored with RCS. In the total population (Figure 3), the overall associations between vitamin A, thiamin, vitamin C, and magnesium and Hb were significant (*p* < 0.05). The level of Hb increased with the intake levels of vitamin A, thiamin, vitamin C, and magnesium. The intake levels of thiamin and vitamin C showed linear relationships with Hb (*p* for non-linear > 0.05), while vitamin A and magnesium showed a non-linear relationship with Hb (*p* for non-linear < 0.05). Although the inverted U-shaped association of vitamin A and magnesium with Hb indicated that the *β* value increased initially and then declined toward the end, the β values remained above 0.

Among female participants (Supplementary Figure S2), the overall associations between vitamin A, thiamin, vitamin C, and selenium and Hb were significant (*p* < 0.05). The intake levels of thiamin, vitamin C, and selenium showed linear relationships with Hb (*p* for non-linear > 0.05), whereas vitamin A showed a non-linear relationship with Hb (*p* for non-linear < 0.05). No statistically significant associations were observed among male participants (Supplementary Figure S3).

### 3.5 WQSR analysis

Two indices were constructed by incorporating constraints in both the positive and negative directions of the effect to reflect the joint effects of micronutrients. No significant associations were observed in either direction for Hb and anemia (Supplementary Table S1).

## 4 Discussion

The present study provides important up-to-date evidence on the current burden of micronutrient intake inadequacies. In addition, the individual and combined effects of micronutrients on Hb and anemia were investigated. Overall, we observed particularly high inadequacies in the intake for riboflavin, vitamin E, calcium, and thiamin, with prevalence rates exceeding 80% (Figure 2). In the female population, dietary intakes of vitamin A, thiamin, vitamin C, and selenium were positively associated with Hb levels (Supplementary Figure S2). Moreover, higher dietary intakes of selenium and thiamin were significantly associated with a decreased risk of anemia among female participants (Table 4).

Simone Passarelli et al. (20) evaluated dietary micronutrient inadequacies across populations aged 0 to ≥80 years in 31 countries spanning Sub-Saharan Africa, South Asia, Latin America and the Caribbean, North America, the Middle East and northern Africa, East Asia and the Pacific, and Europe and central Asia. Compared with the previous study (20), our estimates generally indicated a higher prevalence of inadequate intake for riboflavin (88.1% vs. 55%), vitamin E (87.6% vs. 67%), calcium (81.6% vs. 66%), thiamin (81.1% vs. 30%), vitamin A (74.3% vs. 48%), magnesium (62.8% vs. 31%), vitamin C (61.3% vs. 53%), zinc (43.2% vs. 46%), and niacin (23.4% vs. 22%). In contrast, intake inadequacies for iron (13.3% vs. 65%), iodine (7.06% vs. 68%), and selenium (3.15% vs. 38%) were not as severe (Figure 2). The higher intake inadequacies for most micronutrients observed in this population underscore the need for urgent attention.

In the present study, older age, female sex, and rural residence were positively associated with anemia (refer to Table 3), consistent with findings from previous investigations (27, 28). Age-related physiological changes, such as reduced erythropoietin production by the kidneys or decreased red cell production in the bone marrow, may contribute to the higher risk of anemia (29). In our results, over 10% of participants were affected by anemia, with female participants exhibiting a higher susceptibility. This disparity may be attributed to the biological vulnerability associated with iron loss during menstruation in the reproductive age, as well as dietary inadequacy. Previous studies have shown that dietary diversity among residents in rural areas in China was low, especially in poorer areas where the diet consisted primarily of grains with inadequate intake of animal-based foods (30, 31), which might be the reason for the positive association between living in rural areas and anemia. The presence of diabetes is another positive predictor of anemia prevalence in the present study (refer to Table 3). Evidence from a follow-up study of the UK Biobank also showed that diabetes was positively associated with anemia (32). Diabetes-related chronic hyperglycemia tends to induce a hypoxic environment in the renal interstitium, which results in the impaired production of erythropoietin by the peritubular fibroblasts and subsequent anemia (33). In addition, high income, moderate physical activity, and being overweight or obese were negatively associated with anemia (refer to Table 3). Anemia serves as an indicator of socioeconomic disadvantage because it is inversely related to the socioeconomic status of households in developing settings. People with low income could not receive adequate nutrition through dietary diversification and intake of foods with high iron bioavailability (34). Physical activity might have a positive association with reduced symptoms of anemia (35). It has been demonstrated that physical activity can enhance hemoglobin and red blood cell mass through stimulating erythropoiesis and improving the hematopoietic microenvironment in the bone marrow (36). The relationship between anemia and overweight or obesity remains controversial. In contrast, some studies have shown that, among individuals with high BMI, the adipose tissue releases hepcidin, which causes iron retention within the spleen and the liver, thus reducing uptake by tissues and erythropoietic cells of the body (37, 38). Obesity could impair iron metabolism and is associated with an increased risk of anemia (39, 40). In contrast, some previous studies have found a negative association between overweight and obesity and anemia (41, 42), and the results of this study align with this observation. Furthermore, a study explored the relationship between anemia and iron status and various body size phenotypes in the adult Chinese population from CHNS and found an inverse association between BMI levels and anemia, as well as positive associations between higher serum ferritin and transferrin levels and obesity and overweight (43). The dietary pattern, characterized by a heavy intake of red meat, was positively associated with obesity (44, 45), and obese individuals are more likely to adhere to this red meat-rich pattern (46, 47). Since meat, especially red meat, is the primary contributor of heme-iron absorption in the Chinese population (48), greater red meat intake among overweight and obese individuals could enhance heme-iron intake, thereby reducing the risk of anemia. This may explain why overweight and obesity were negatively associated with anemia prevalence in the present study. Cigarette smoking could cause increased hemoglobin levels and secondary polycythemia (27, 49), a phenomenon likely mediated by exposure to carbon monoxide, which markedly reduces oxygen-carrying capacity. To compensate for this reduced oxygen delivery, smokers typically maintain higher Hb concentrations compared to non-smokers (50). Similar to the previous study (27), we found that current smokers were less likely to have anemia (refer to Table 3). Nevertheless, the health hazards of smoking cannot be ignored.

In the present study, we found that the level of Hb increased with the intake level of vitamin A, thiamin, vitamin C, and selenium among women (refer to Supplementary Figure S2). Moreover, selenium and thiamin were negatively associated with the risk of anemia among women (refer to Table 4). A previous study had indicated that vitamin C could regulate transferrin iron uptake and that vitamin C deficiency might induce anemia (51). Dietary intervention, including increased intake of vitamin C, has been recommended for anemic female patients (52). Xie et al. investigated associations between multiple trace metals (Ni, Co, Mn, Se, and Mo) and anemia in US adults from the National Health and Nutrition Examination Survey (NHANES) study and found that selenium was negatively associated with the risk of anemia (53). Another study among older Italians also found that low plasma selenium was a long-term independent predictor of anemia (54). Selenium is a potent nutritional antioxidant, and it manifests its biological functions by being incorporated into selenoproteins, such as glutathione peroxidase (GPx). A modest decrease in the levels of GPx could be partly responsible for the peroxidation of red blood cells, which results in anemia (55). There are relatively few epidemiological studies exploring the association between thiamine and anemia, yet existing studies have shown that thiamine is crucial for human health (56). Vitamin A is essential for normal erythropoiesis. It indicates that, in vitamin A deficiency, iron mobilization is impaired, and that this mineral accumulates in the liver and the spleen, making it less available for erythropoiesis (57).

This study has several strengths. One is that the study participants recruited to estimate micronutrient intake levels and to evaluate their relationship with anemia were derived from a relatively large health survey. Another is that a wide range of covariates was adjusted to bolster the robustness and reliability of the results. However, the study has some limitations that should be considered. First, the causal association between micronutrients and Hb or anemia could not be identified due to the study’s cross-sectional design. The simultaneous collection of exposure and outcome data cannot establish the temporal sequence of events, which is a fundamental prerequisite for determining causality. Second, the dietary intake data in this study were collected using the consecutive 3-day 24-h dietary recall method, a widely used approach in population-based nutritional epidemiological studies (14). During data collection, trained investigators conducted face-to-face interviews with participants to record detailed information on all foods and beverages consumed (including main meals, snacks, and condiments). Special attention was paid to food types and intake amounts (estimated using standard household measuring tools such as bowls, spoons, and scales to improve accuracy). However, recall bias may exist, which could affect the accuracy of the data. Third, the 3-day 24-h dietary recall method used in this study was not validated in our study population. However, since the 3-day 24-h dietary recalls method used in this study is consistent with that of the CHNS, a nationwide health and nutrition survey project (58), the accuracy of the 24-h dietary recall designed to assess nutrient intake has been previously validated (58, 59). Compared with the gold standard (household inventory) used in China, for the modified 3-day 24-h dietary recall method employed in the CHNS, the ratios of selected nutrient intake measured by the two methods were remarkably close (59). Fourth, a limitation of this study is the absence of nutrient biomarker measurements, which prevents us from linking reported dietary nutrient intake to actual *in vivo* nutrient status (e.g., absorption, metabolism, and storage) and thus weakens the robustness of causal inferences regarding nutrient-anemia associations. Fifth, the original survey did not collect data on menstrual blood loss and menopausal status—two factors closely tied to anemia risk, as menstrual blood loss directly affects iron reserves and menopausal status reshapes hormonal regulation of nutrient metabolism—leaving residual confounding unaddressed in our statistical models.

## 5 Conclusion

Micronutrient intake inadequacy was generally high in the Chinese study population, and public health interventions should be implemented to effectively address the identified deficiencies. Among the micronutrients examined, vitamin A, thiamin, vitamin C, and selenium showed a significantly positive association with Hb level in the female population. Selenium and thiamin showed a significantly protective effect against anemia among females. Individuals with anemia should therefore be assessed for multiple micronutrient levels and then treated appropriately. This study underscores the necessity for a refined approach to anemia, moving beyond iron to consider multiple micronutrient deficiencies. Key future directions include elucidating the biological mechanisms of non-iron micronutrients, developing and testing integrated supplementation strategies, and optimizing food fortification programs for precision prevention.

## Data Availability

The raw data supporting the conclusions of this article will be made available by the authors, without undue reservation.
