# Supplementary material for: Comprehensive assessment of dietary micronutrient profiles and their effects on hemoglobin levels and anemia: provincial nutrition and health monitoring
Source: Front Nutr. 2025 Nov 26;12:1638705. doi: 10.3389/fnut.2025.1638705 (PMC12689416; doi:10.3389/fnut.2025.1638705)
Supplement: Supplementary file 1 [file Table_1.docx]

Table S1: Overall effects of micronutrient mixtures on hemoglobin levels and anemia based on weighted quantile sum regression analysis.

|  | WQS indices direction | %Δ (95%CI) |
| --- | --- | --- |
| Hb | Positive | 0.06% (-0.30%, 0.42%) |
|  | Negative | -0.06% (-0.41%, 0.29%) |
| Anemia | Positive | 4.56% (-4.89%, 14.0%) |
|  | Negative | 6.81% (-4.82%, 18.4%) |

Adjusted by age, sex, energy of intake, living area, education level, income, smoke, physical activity, BMI, hypertension, diabetes, use of dietary supplements, and Hs-CRP level.


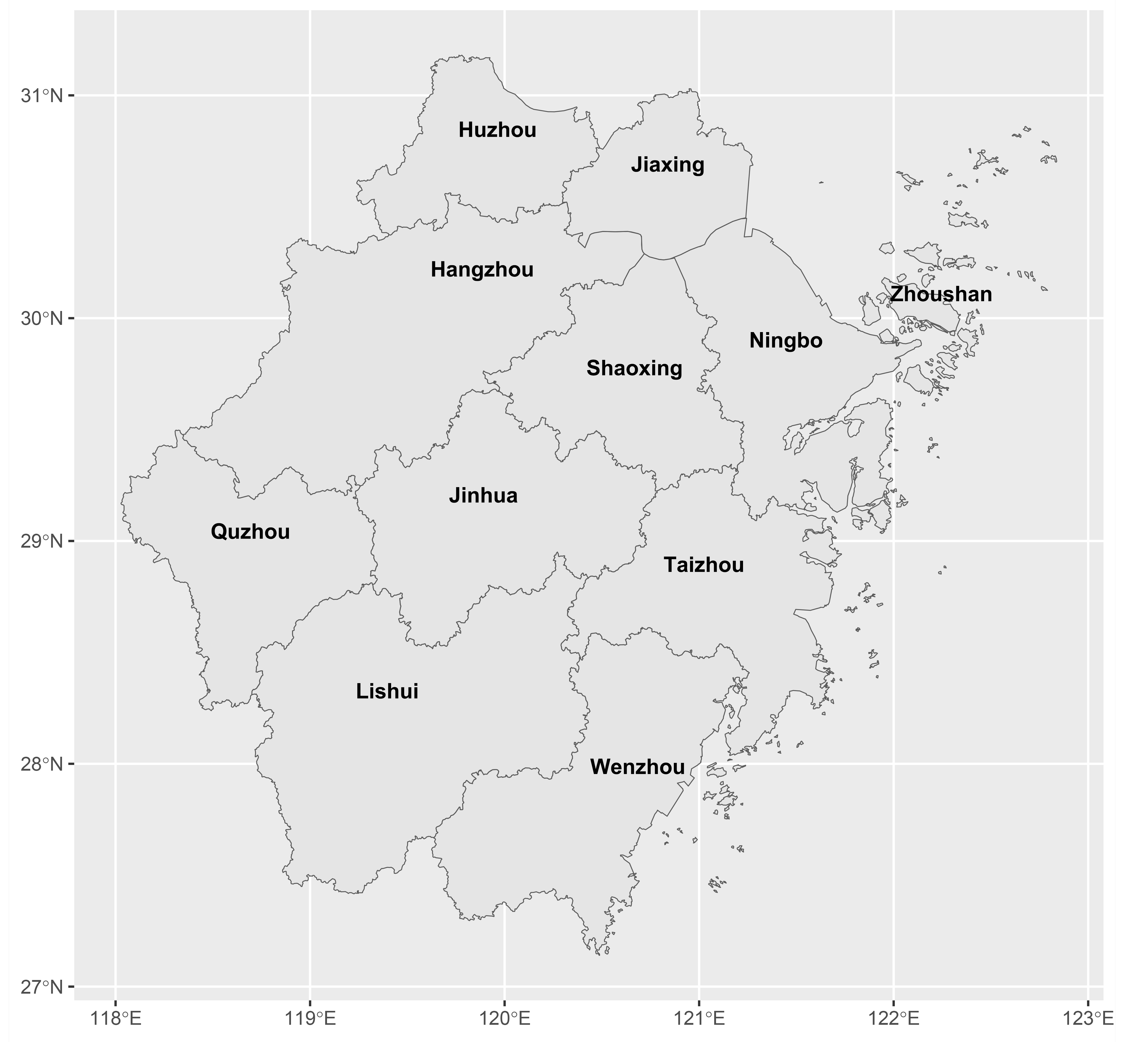


Figure S1. Geographical map of 11 cities in Zhejiang Province, indicating the regional coverage of the study population involved in the micronutrient and anemia analysis.


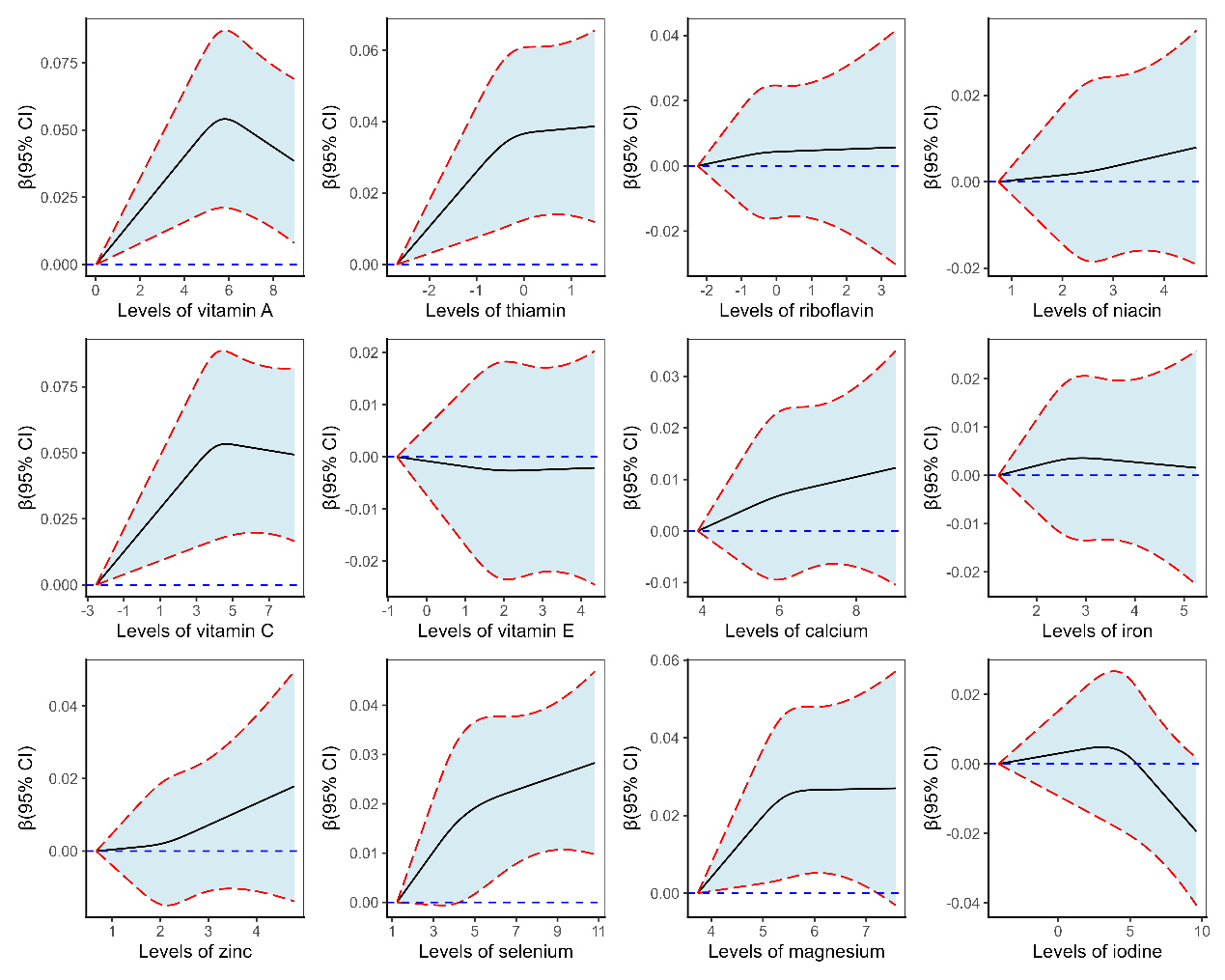


Figure S2: Restricted cubic spline (RCS) analysis depicting the association trend between micronutrient levels and hemoglobin levels in female participants.

Adjusted by age, energy of intake, living area, education level, income, smoke, physical activity, BMI, hypertension, diabetes, use of dietary supplements, and Hs-CRP level.


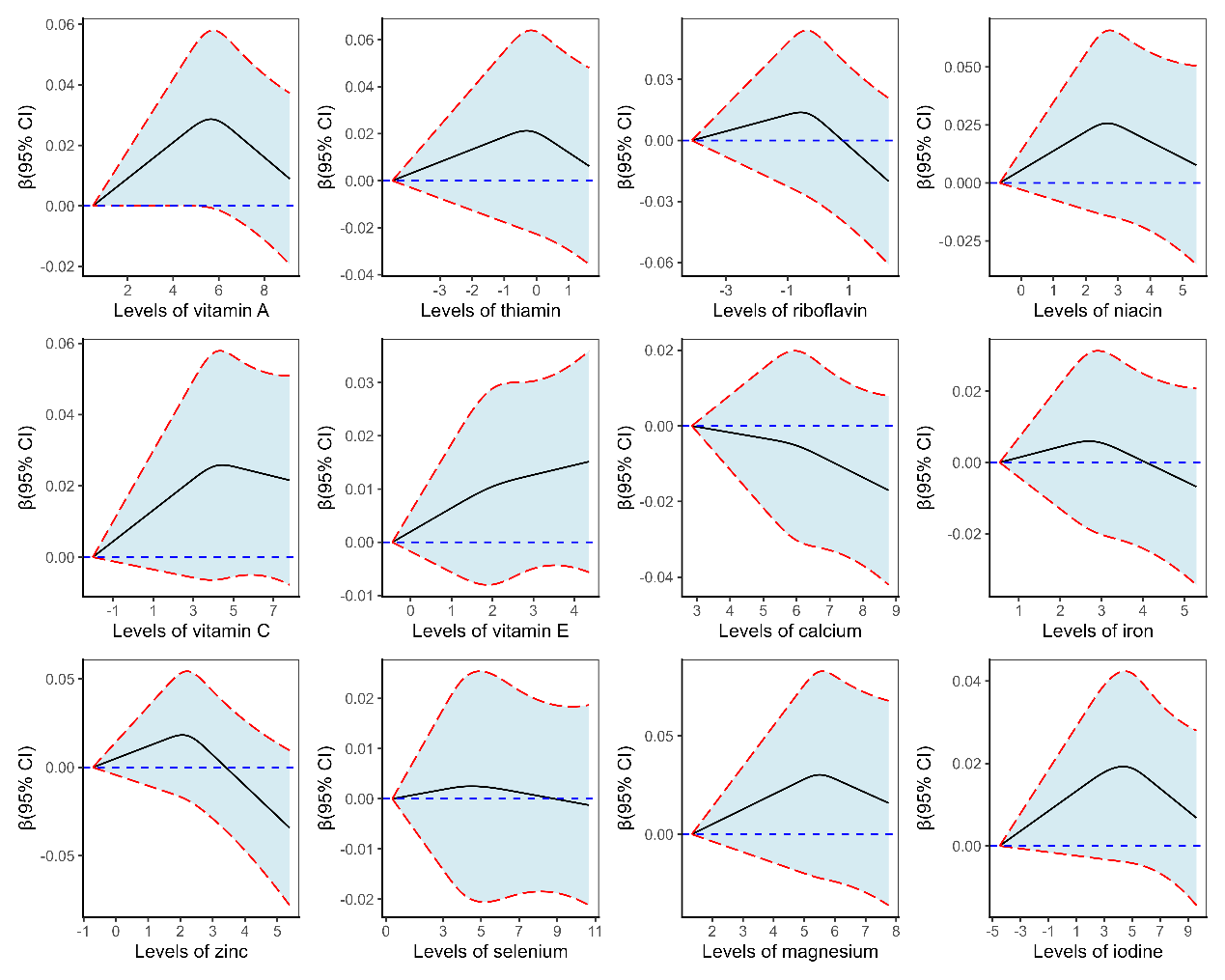


Figure S3. Restricted cubic spline (RCS) analysis depicting the association trend between micronutrient levels and hemoglobin levels in male participants.

Adjusted by age, energy of intake, living area, education level, income, smoke, physical activity, BMI, hypertension, diabetes, use of dietary supplements, and Hs-CRP level.
